# Softball Petechiae: A Novel Cutaneous Finding in a Patient Participating in Post-Exercise Massage

**DOI:** 10.7759/cureus.777

**Published:** 2016-09-12

**Authors:** Talayesa Buntinx-Krieg, Jeffrey Greenwald

**Affiliations:** 1 University of Central Florida College of Medicine; 2 Dermatology, University of Central Florida College of Medicine; 3 The Dermatology Group, P.A.

**Keywords:** traumatic petechiae, petechial rash, petechial eruption, traumatic purpura, post-exercise massage, sports rash, softball petechiae, softball massage, sports trauma, softball

## Abstract

We report a case of a 39-year-old healthy male presenting with an eruption consisting of evenly spaced, well-circumscribed, round, petechial macules over a discrete region on his back. A detailed history revealed that the man participated in a high-intensity combination workout routine and post-exercise massage. He reported using a regulation-sized dimpled softball in order to massage the musculature of his back. A diagnosis of traumatic petechiae was established.

A growing fitness culture encouraging high-intensity training and post-exercise massage coupled with the high costs of professional masseuse services has led to the increased use of self-massage techniques using both traditional and non-traditional massage equipment. The topography of this equipment and the rise in post-exercise self-massage may lead to an increase in traumatic rashes of varying clinical and cosmetic significance.

## Introduction

Healthy patients may present with strange dermatologic findings, and a thorough patient history and keen observational skills are required to determine the cause. However, the history is often riddled with exposure to multiple potential culprits, which can complicate the diagnostic process. While some cutaneous eruptions are nonspecific in nature, oftentimes there are characteristics of the eruption that point the clinician in a particular direction. A thorough evaluation of the primary lesions, their location, configuration, texture, distribution, and color are key in deducing the correct diagnosis [1]. At times, the cutaneous eruptions are the result of patient behavior including but not limited to post-exercise massage techniques. Literature regarding the cutaneous manifestations secondary to post-exercise massage is sparse. Informed consent was obtained from the patient for this study.

## Case presentation

**Figure 1:**
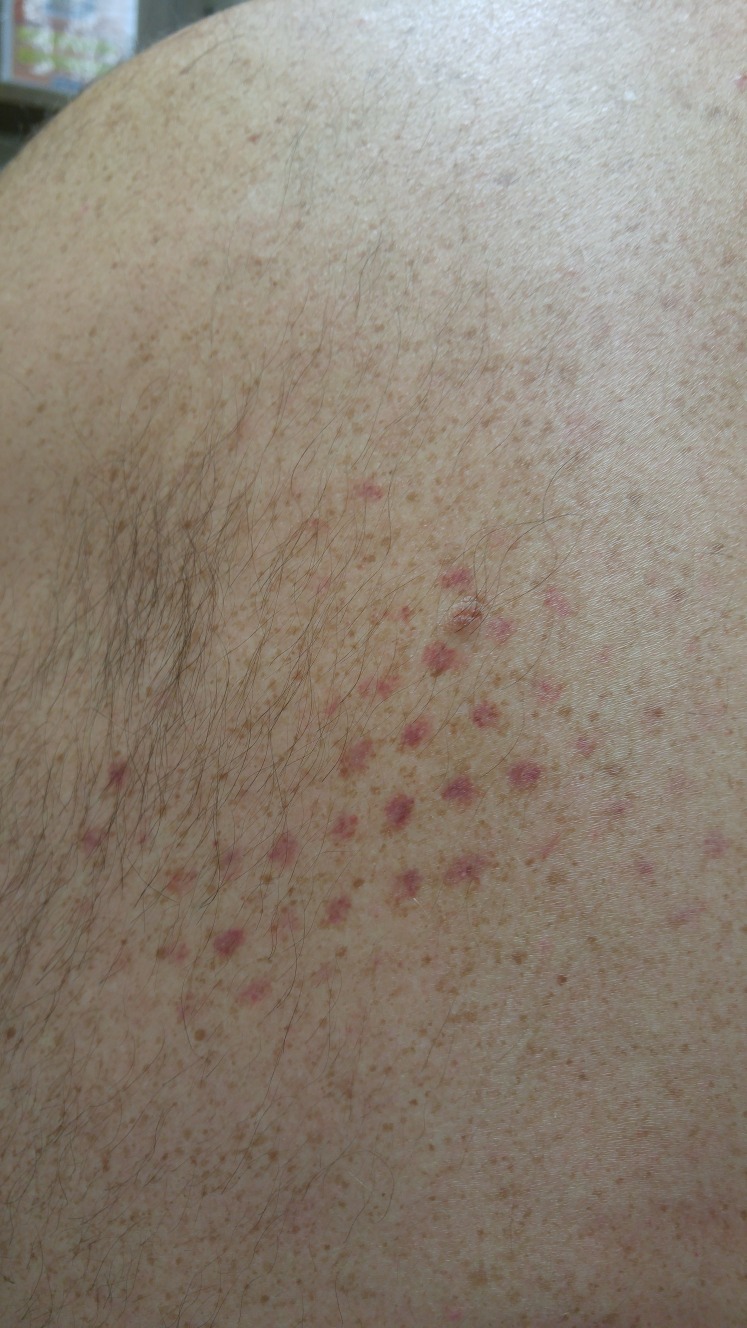
Traumatic Petechiae Evenly spaced, well-circumscribed, round, non-blanching macules observed on the back in a region occupying an area of 10 cm x 15 cm.

A healthy 39-year-old male presented to the dermatology clinic for his routine annual skin exam with no complaints. He mentioned exercising regularly, incorporating high-intensity combinations of cardiovascular and strength training into his daily routine. His past medical history was significant for plantar warts (verruca plantaris) and a melanoma in situ on the right side of the neck that was completely excised four years prior to presentation. His only medication was sertraline. His past social and family histories were noncontributory. On examination, his skin showed signs of chronic sun exposure with a diffuse tan appearance and multiple hyperpigmented macules over the nose, cheeks, shoulders, forearms, hands, and lower extremities. On his left superior back, evenly-spaced, well-circumscribed, round, petechial macules were observed occupying an area of roughly 10 cm x 15 cm as seen in Figure 1.

Upon questioning regarding the lesions, the patient recalled having used a regulation-sized dimpled softball to massage his back following his exercise routine. He described placing the softball between his back and a wall and turning his trunk side-to-side to massage the musculature of the back. A diagnosis of trauma-induced petechiae was established.

## Discussion

This patient presented with skin lesions that were initially perplexing; however, multiple clues in the history and physical appearance of the eruption helped make the diagnosis. When cutaneous findings lead the clinician to suspect an external source, it may be useful to question the patient about the potential behaviors and triggers. In this case, the patient revealed a history of high-intensity combination fitness training and the use of a post-exercise massage as a part of his regimen. There have been reports of sports-related traumatic cutaneous findings induced by contact with equipment and other players; however, there is no information regarding cutaneous findings associated with the post-exercise massage [2-5].

Massage therapy has been shown to reduce inflammation and promote mitochondrial biogenesis following exercise-induced muscle damage [6]. Nevertheless, a meta-analytic review of massage and performance recovery revealed that the effects of post-exercise massage produce a small benefit, improving post-exercise fatigue in certain instances (short-term recovery following intensively mixed training), particularly among the untrained individuals. The authors concluded that the evidence was not sufficient to justify the widespread use of post-exercise massage [7]. Despite lacking evidence, a growing fitness culture in the United States and the development of multiple novel strength and conditioning programs has led to an increase in the use of post-exercise massage in addition to other alternative post-exercise therapeutic practices. Furthermore, the high costs associated with professional masseuse services have also led to an increase in self-massage techniques using both traditional massage equipment and non-traditional equipment including lacrosse balls, cylindrical pieces of wood, and in this case a softball.

One alternative post-exercise therapeutic practice that has been described in the literature is known as cupping. It is practiced in Asia, the Middle East, Latin America, Eastern Europe, and with increasing frequency in the United States. The cupping involves the application of a heated cup to the skin, and as the air cools, negative pressure is generated leading to a suction force. This practice has been associated with circular erythema, ecchymoses, and pupura secondary to the superficial blood vessel damage in the papillary dermis [8].

The use of both traditional and non-traditional massage equipment may produce cutaneous findings corresponding to the specific topographical features lasting for variable periods of time. The lifespan of these lesions is dependent on the amount of pressure between the object and the skin, the structural integrity of the cutaneous architecture and underlying vasculature, and potential hypersensitivities to materials in the massage equipment. Interestingly, there have also been reported cases of localized psoriasis secondary to Koebnerization induced by the cupping therapies [9-10]. In this case, the dimpled topography of the softball used for the massage was responsible for the development of a petechial eruption.

Given the traumatic nature of the lesions described in this case report, the treatment was centered on patient education. This particular patient was not bothered by his lesions but could have avoided additional lesions by using massage equipment with a smooth surface.

## Conclusions

When cutaneous eruptions have a configuration and distribution that suggests an external cause, patient behavior patterns might be the culprit. Traumatic petechial/purpuric eruptions may become a more common finding, particularly among healthy individuals engaging in regular exercise and post-exercise massage. Patients should be educated about the cause of the eruption and how to avoid producing more lesions by changing their massage equipment or technique. Furthermore, patients with dermatologic diagnoses that are prone to Koebnerization should be warned that these practices may exacerbate their condition.
